# Microstructural Characterization of Porous Clay-Based Ceramic Composites

**DOI:** 10.3390/ma12060946

**Published:** 2019-03-21

**Authors:** Lorena Freitas Dutra, Monica E. Freitas, Anne-Cécile Grillet, Nathan Mendes, Monika Woloszyn

**Affiliations:** 1 Department of Mechanical Engineering, Engineering Faculty, Pontifical Catholic University of Paraná, Imaculada Conceição Street, Curitiba 80215-901, Brazil; nathan.mendes@pucpr.br; 2 Univ Savoie Mont Blanc, CNRS, LOCIE, F-73000 Chambéry, France; anne-cecile.grillet@univ-savoie.fr (A.-C.G.); monika.woloszyn@univ-smb.fr (M.W.); 3 Laboratório de Inclusões Fluidas e Metalogênese, CDTN-CNEN-Centro de Desenvolvimento da Tecnologia Nuclear, Comissão Nacional de Energia Nuclear, Caixa Postal 941, Belo Horizonte 30123-970, Brazil; monicaef@gmail.com

**Keywords:** microstructural characterization, clay-based materials, pore size distribution

## Abstract

Clay-based materials are the most traditional components of buildings. To improve their performance in a sustainable way, agents can be mixed to fired clay acting as a pore-forming factor. However, firing temperatures highly influence their microstructure which is closely linked to a material’s final performance as a ceramic block. To highlight the influence of the firing temperature on microstructure, and more specifically on the pore size distribution of clay-based materials, three innovative porous materials were manufactured. These materials were produced by mixing clay and pore-forming agents. They were characterized by optical and scanning electronic microscopy, x-ray diffraction, mercury intrusion and nitrogen adsorption. These techniques allow the phase identification of materials, show sample microstructure and quantify the pore size distribution at different scales. Furthermore, geometric parameters of sample microstructure such as grain diameter and roundness are estimated by using computer software. To conclude, results provide an enlightenment about the influence of material microstructure on the pore size distribution at two firing temperatures. These results can be useful to allow the tune of porous characteristics and, therefore, contribute to the production of more sustainable construction materials.

## 1. Introduction

Some researchers highlighted an influence of firing temperature on clay materials’ microstructures, as well as on physical and mechanical properties [1,2,3,4,5,6] while others analyzed pore size distribution of natural [7,8] and fired clays [9,10]. Mineralogical composition and particle size link was also studied by [11]. Yet, papers correlating firing temperature, microstructure and pore size distribution in fired clays are few. To fill this gap, this paper investigates the influence of composition and firing temperature on the microstructural evolution of three clay-based materials and links the referred properties to pore size distribution.

Fired clay is a common building material [12] and Brazil is one of the world’s largest producers of clay-based ceramic [13]. Different types of additives can be incorporated to fired clay in order to improve its properties [14]. For instance, these additives act as pore-forming agents increasing thermal and acoustical insulation capacity while maintaining load bearing capacity, reducing bulk density and improving hygric performance [15,16]. Additives can also be incorporated to clay to lower the sintering temperatures and, thereby, realize energy saving estimated at between 1.2 and 5.5 m${}^{3}$ of natural gas gross per tonne fired product [17]. In this case, low-cost and easily found mineral absorbents were chosen to act as pore-forming agents such as: perlite and attapulgite.

Perlite is a glassy volcanic rock that can expand up to 30 times of its volume when fired up to 1200 °C [18]. It is usually used in thermal or acoustic insulation, water retention and soil aerator for agriculture and horticulture due to its high porosity and low density [19]. Commercial palygorskite (attapulgite) has many applications in different areas such as catalysis, agriculture and environmental protection also due to its high adsorption capacity [20]. The attapulgite provided for this work has been sold as a road absorbent granule absorbing oil, lubricants, flammable and other pollutants.

The size and the distribution of pores in a material is very important to understand their hygric, mechanical and thermophysical behavior. To find out whether pore size distribution affects microstructural properties with firing temperature, a detailed microstructural characterization of clay-based materials has been performed. Results from an experimental campaign that includes elemental and phase composition, microstructure morphology at micro and nanoscales and pore distribution quantification are presented. For this purpose, nitrogen adsorption technique based on the Brunauer–Emmett–Teller (BET) theory [21] is very useful to access pore information but it is limited to small pores. In order to have more accurate data of larger pores, the mercury intrusion porosimetry (MIP) technique is also used. It is an analogous process with $N_{2}$ adsorption but it uses mercury as the pore filler instead of nitrogen. However, the MIP method tends to underestimate the volume of the smallest pores due to the ink-bottle-shaped pores which means that large pores with small openings are identified as having a small diameter making the BET method more appropriate to measure small pores. Therefore, a combination of MIP and $N_{2}$ gas adsorption can provide information for the entire pore-size range. Typically, the pore diameter determination range of high-pressure mercury porosimetry is 10–300 μm and nitrogen adsorption is 1–100 nm [22]. Elemental composition was given by energy-dispersive X-ray spectroscopy (EDS). Nevertheless, a ceramic material is not only governed by its chemical composition, but also by its crystalline structure. For instance, two ceramics with an identical composition but a different crystal structure may behave very differently [23]. Thus, X-ray diffraction (XRD) aided to characterize phase composition. Morphology from a scale of micrometers was obtained with optical microscopy (OM) and analyzed with the software Quantikov [24]. In addition, structures from nanometers were illustrated with the images from scanning electron microscopy (SEM).

## 2. Materials

Pore-forming agents perlite and attapulgite were obtained from Eurosorb CO., in Thourotte, France. Clay was provided from a tile and brick company located in Curitiba, Brazil. Samples were prepared by mixing clay with 20% in mass of absorbents. Clay was previously dispersed in water by mixing during 10 min. All compositions were mixed during additional 20 min. The mixtures were then transferred to the mold conferring their final shape. The initial cure was carried out at controlled temperature (30 °C ± 2 °C) for 48 h. After this period, samples were removed from the molds and fired at 105 °C for the following 24 h. After curing, samples were sintered in a furnace at the firing temperatures of 800 °C and 1000 °C for 1 h. The fabrication conditions of the samples were based on the process that is commonly used to produce bricks and tiles as well as the choice of these two firing temperatures.

## 3. Methods

X-ray diffraction (XRD) analyses were performed using a Rigaku D/MAX Ultima diffractometer from CDTN (Centro de Desenvolvimento da Tecnologia Nuclear, Brazil). The samples were analyzed as powders after a heat treatment at 800 °C and 1000 °C. Clay of the base material was dried and crushed. Absorbents (Attapulgite and Perlite) were also crushed and analyzed for initial composition identification, which enables the examination of absorbents influence when they are mixed to clay and sintered. Data were collected on a range of 2θ from 4° to 80° at a scan speed of 2°/min, current intensity of 30 mA and voltage of 40 kV and using a CuKα radiation source (λ = 1.5405 Å). The identification of the mineral phases was carried out using Jade 0.9/MDI software and PDF2 database.

Optical microscopy observations were conducted using a polarized microscope (Leica 4500) with a image capture system from Fluid Inclusions and Metallogenesis Laboratory at CDTN (Centro de Desenvolvimento da Tecnologia Nuclear) and samples were prepared as polished thin sections. The main textural features observed in the optical microscopy were analyzed and measured using the software Quantikov. It provides the following microstructural parameters: the area, the perimeter, the length, the diameter, roundness and a statistic distribution of the grain size and phases in the microstructure of the material [24].

The microstructural study was complemented by the scanning electron microscope (Vega3, Tescan) from Materials Technology and Manufacturing Processes Laboratory at PUCPR, equipped with an energy dispersive X-ray spectroscope (EDS). The polished thin sections were gold coated in the Quorum Q150R ES rotary-pumped coating system. The images were obtained using secondary electron (SE) detector, operated at a 10.0 kV accelerating voltage for morphology visualization.

Pore size distribution was obtained by two methods: nitrogen adsorption and mercury intrusion porosimetry (MIP). The NOVA 2000e, Quantachrome from Ecomateriales laboratory at UANL (Universidad Autónoma Nuevo León, Mexico), is a surface area analyzer equipment that was used to obtain pore size distribution of samples using the Brunauer–Emmett–Teller (BET) theory [21]. Nitrogen was the gas used as adsorbate since it does not react chemically with the material surface allowing the quantification of surface area and pore size. Besides of pore size distribution, BET theory allows the calculation of materials specific surface. Mercury intrusion porosimetry (MIP) from École des Mines d’Alès in France was used to access larger pores that BET technique cannot measure. Therefore, MIP combined with nitrogen adsorption provided the complete range of pore size distribution as well as pore shape and pore surface area.

## 4. Results and Discussion

### 4.1. Phase Identification and High Temperature Behavior

The characterization of the phases was carried out initially through XRD for the identification of the very fine phases and, mainly, the clayey type. However, quantification of the phases by this method was disregarded. Since the high intensities of the quartz and microcline peaks are related to the degree of crystallinity of these minerals when compared to the clay minerals, they can not be attributed to the proportion of these minerals.

**Figure 1:**
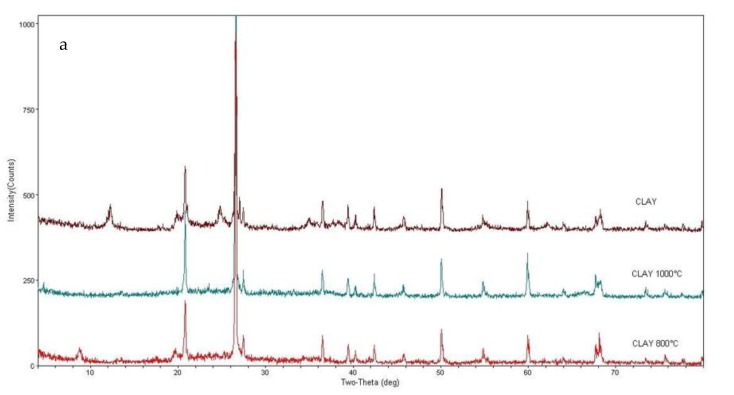

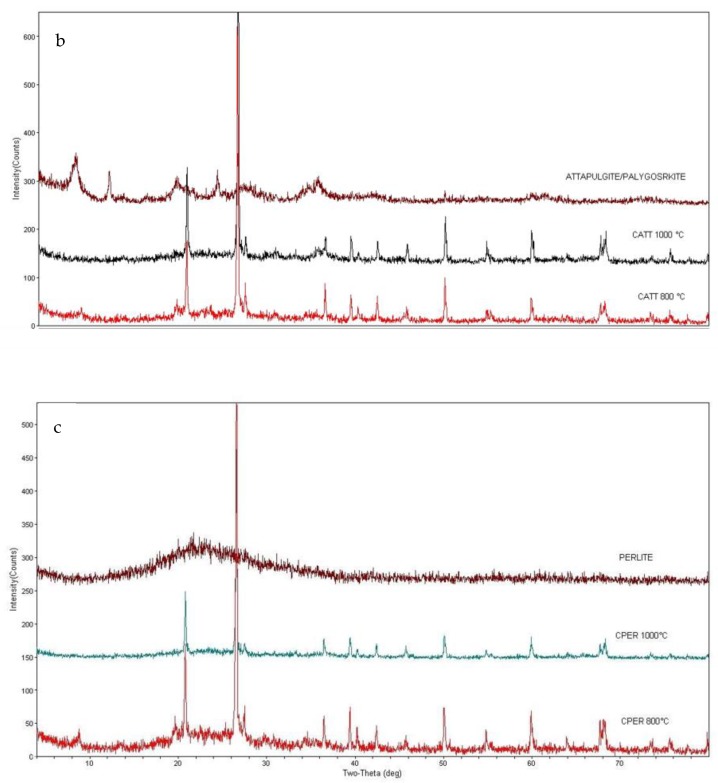
X-ray pattern to show the phase evolution of (**a**) clay, (**b**) clay with attapulgite and (**c**) clay with perlite when firing temperature is increased. Qtz = Quartz, Kaol = Kaolinite, Mc = Microcline, Ms = Muscovite, Lz = Lizardite, Pal = Palygorskite (abbreviations from [32]).

The mineralogical composition of the clayey material consists predominantly of kaolinite, quartz, microcline and muscovite, characteristic of kaolinic clays. Figure 1a shows a comparison of the XRD spectra generated for the initial clay and the fired ceramic material at 800 °C and 1000 °C denoting a variation of the phases with the sintering temperature of the material. The kaolinite peaks disappear with the heating process which was expected since kaolinite loses its (OH) lattice water between 500 and 600 °C transforming to a new phase known as metakaolinite [25]. The XRD pattern of the metakaolinite corresponds to that of an amorphous material [26]. Therefore, at 800 °C kaolinite and metakaolinite peaks are not observed. However, muscovite peaks were observed at this temperature. At 1000 °C, only the peaks of the quartz and microcline remained.

In the ceramic material with the addition of attapulgite, the comparison of the diffraction results in the attapulgite pellets and the fired material at 800 °C and 1000 °C showed that the influence on the mineralogical composition is predominantly of the clay base material (Figure 1b). The attapulgite granules were crushed and the powder was analysed by XRD. The attapulgite pellets are essentially composed of: paligorskite, lizardite and muscovite/illite and quartz. In the ceramic material with attapulgite sintered at 800 °C, the paligorskite and lizardite peaks do not appear, remaining only the muscovite, quartz and microcline. At 1000 °C, only peaks of quartz and feldspar are observed, as in the base material.

Paligorskite is the main constituent mineral of attapulgite granules and its sorption and high natural porosity characteristics are dependent on the outer surface and the size of its molecular channels [27]. The morphological and structural characteristics of paligorskite [28,29] and the variations that occurred during the heat treatment processes [27,30,31], have been exhaustively studied. In this work, we found that the paligorskite undergoes drastic structural variations during heating. At 120 °C half of the coordinated water is lost and the structure folds during the removal of 50–65% of the structural water. The disappearance of palygorskite is consistent with structural variations occurring with the increasing of firing temperature, where dehydration and dehydroxylation is complete between 400 and 500 °C, depending on the rate of heating [27]. The temperature to which these samples were submitted to is much higher than the one shown in all the experimental works. The transformation of paligorskite in other phases with the heating will release water and generate variation in the internal structure of the granules, which may facilitate the start of the melting, justifying the characteristics of the diffractograms, with lower peak definition.

In the perlite-containing ceramic material, the influence of the additive can still be observed at 800 °C, but at 1000 °C, only quartz and feldspar remained in the base material. The perlite forms a broad peak with a maximum elevation of 2θ at 22°. At 800 °C, the diffractogram shows background variation, coincident with the perlite curve. Muscovite, quartz and microcline peaks were also observed, as in the base material. At 1000 °C, there is no more influence of perlite, nor does it act as an amorphous material (Figure 1c).

### 4.2. Microstructure Morphology

#### 4.2.1. Clay

**Figure 2:**
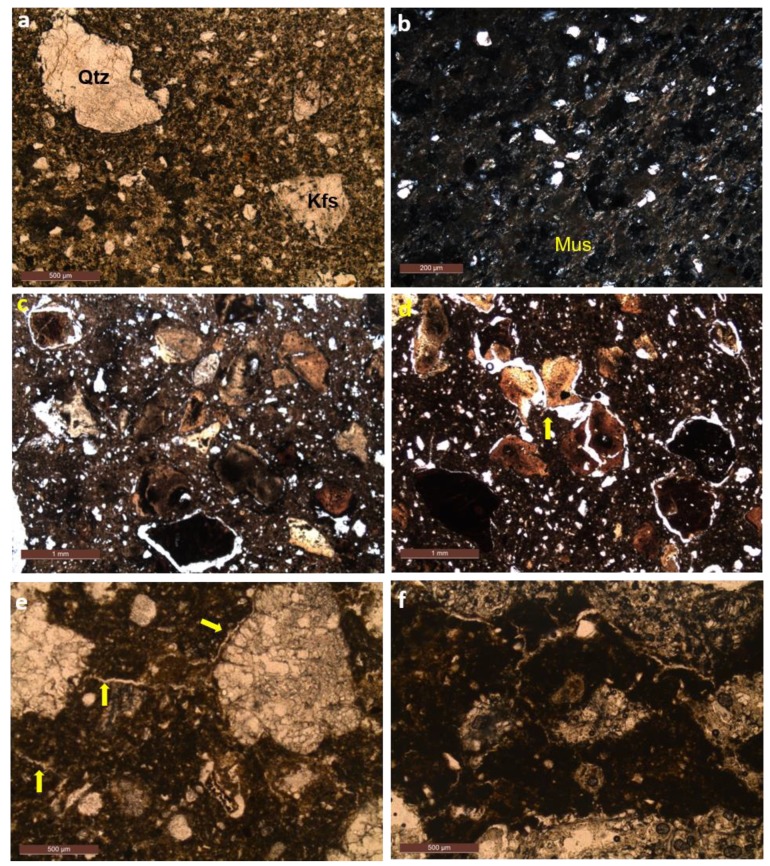
Photomicrographs of the clay samples without addition of absorbents showing the main characteristics of the clayey base material fired at 800 °C: (**a**) larger fragments of quartz crystals (Qtz) and feldspar (Kfs) supported by a clay matrix with small crystals of quartz and feldspar. Samples fired at 1000 °C: (**b**) darker clay matrix with fragments of rock (R × F) and quartz agglomerates (natural light). Samples containing attapulgite particles fired at 800 °C: (**c**) general characteristic of the sample showing the different behavior of the attapulgite particles with the heating, generation of a crack porosity around some particles and the presence of fractures in the particles of attapulgite and particles with darker central portion indicating initiation of a fusion process (natural light) and (**d**) detail of the thicker coronas and that the firing process generated the fracture and fragmentation of some attapulgite particles and formation of channels to a lesser extent fired at 1000 °C. (**e**) small channels connecting the perlite particles fired at 800 °C and (**f**) formation of channels in greater proportion and size for samples fired at 1000 °C.

Figure 2a,b are representative samples of the base clay material, without the additives. It is characterized by containing fragments of quartz crystals, feldspar and iron hydroxides, and to a lesser extent fragments of rocks, supported by a clayey matrix. The staining of the matrix varies from light beige to reddish, with the darker portions resulting from the presence of colloidal iron hydroxides.

In the sample fired to 800 °C, two granulometric bands of fragments are observed: larger, between 500 and 1000 μm, and small ones, smaller than 50 μm. It was also identified the presence of iron oxide and hydroxide crystals, occurring as subhedral crystals. In the clay matrix, the crystals of quartz and feldspar are distinguishable by optical microscopy and the presence of fine muscovite is highlighted (Figure 2a).

However, in the sample fired at 1000 °C (Figure 2b), the muscovite disappears, the matrix is darker with several types of rock fragments and minerals, in which shapes and sizes are similar to those found in the sample fired to 800 °C. The fragments of quartz and feldspar are more difficult to distinguish through the optical properties, since the twinnings in the feldspar disappear.

#### 4.2.2. Clay with Attapulgite (CATT)

In the samples in which attapulgite particles were added, microcracks appeared around the fragments with the heating process, generating a crack porosity [33]. At 800 °C (Figure 2c), attalpulgite particles exhibit partial fusion features, with light, medium and dark portions. These structures may be rounded or irregular, but generally, the central portions are the darkest. The lighter portions of the attapulgite particles still preserve the optical properties of the original material, however the darker portions become extinct, indicating fusion. Also, some particles are partially fractured, and the opening of these fractures also generate the crack porosity. In the clayey matrix there are still fine muscovite crystals, mainly observed with crossed nicols. The circular crack porosity, similar to a corona, found around some attapulgite particles, may be complete, but it is generally narrow and does not occur in all grains. It does not occur neither around the remaining crystals nor rock fragments of the clayey base material.

In the sample fired to 1000 °C (Figure 2d), it is observed that both the matrix and the attapulgite particles are darker, characterizing the burning of the material. The crack porosity coronas around the attapulgite particle are more abundant and thicker at this temperature. This crack porosity can be interconnected when there is a fracturing of attapulgite particles. To a lesser extent, crack porosity may form irregular channels in the matrix.

#### 4.2.3. Clay with Perlite (CPER)

In the samples with addition of perlite (Figure 2e,f), the main texture formed with the firing process is a crack porosity in the form of channels, which tend to bind one particle to another. At 800 °C (Figure 2e), the perlite still preserves its internal structure, formed by rounded fractures. There are small irregular channels forming that interconnect the perlite particles and the rock fragments and larger crystals of the base material. These channels can be observed even in the cross section of the sample, indicating that it occurs in the three dimensions of the material. At 1000 °C (Figure 2f), the channels are more abundant and thick, but there is a larger interaction with the rock fragments and the iron crystals/oxides/hydroxides.

### 4.3. Microstructural Evaluation

**Figure 3:**
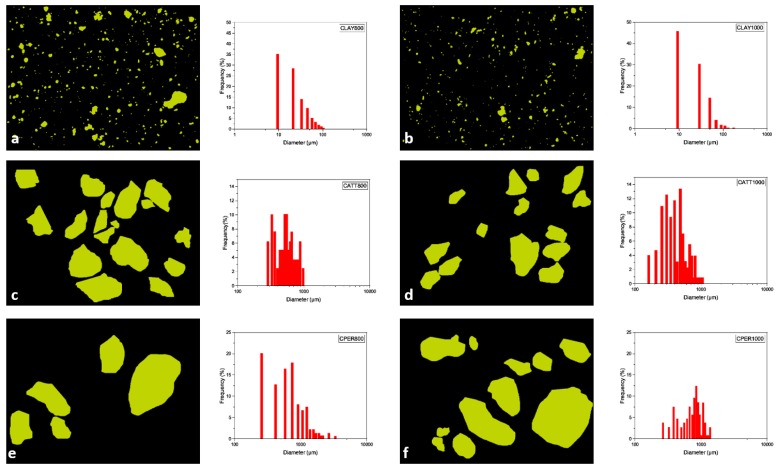
Examples of structures identification and quantification by the software Quantikov. (**a**) Clay fired at 800 °C, (**b**) clay fired at 1000 °C, (**c**) clay with attapulgite fired at 800 °C, (**d**) clay with attapulgite fired at 1000 °C, (**e**) clay with perlite fired at 800 °C, (**f**) clay with perlite fired at 1000 °C.

The observation of the samples by optical microscopy allowed to identify the main structural characteristics of the different compositions and their responses to the heating process. Initially, the characteristics of the base material, clay, were studied to understand its behavior, aiming a comparison pattern with the ceramic material with addition of the pore-forming agents. The textures and microstructures observed in the photomicrographs were treated using the program Quantikov [24] to size the visually observed differences (Figure 3).

The clay material, used as the base reference, has the maximum amount of 5.7% of fragments supported by the clay matrix. These fragments were identified as quartz crystals, microcline, iron oxides and hydroxides and rock fragments. The largest proportion of these fragments is lower than 10 μm: 36% in the sintered sample at 800 °C (Figure 3a) and 46% in the sample sintered at 1000 °C (Figure 3b). Approximately 80% of the fragments are smaller than 50 μm. Larger fragments with a length of approximately 100 μm represent less than 4% of the total. The degree of roundness of these fragments is 0.7, and is not influenced by temperature (Table 1). The matrix remained homogeneous, showing no generation of microstructures observable by optical microscopy.

Samples with attapulgite addition were treated in two steps, initially the particles were measured to evaluate their behavior with the heating process. Subsequently, only the crack porosities observed in the images were measured. In the sample sintered at 800 °C, the attapulgite particles ranged from 256 to 1036 μm, with a bimodal behavior, with a moda at 300 μm and another at 500 μm (Figure 3c). In the sample sintered at 1000 °C, the particles ranged from 165 to 1127 μm (Figure 3d). The decrease in the minimum particle size can be explained by the fracturing and fragmentation of the particles, which occurred with the temperature rise, as observed on the image (Figure 2d). The maximum size may be considered the same, which shows that the melting occurred during firing process did not generate a change in the volume of the particles that remained entire (Table 1).

**Figure 4:**
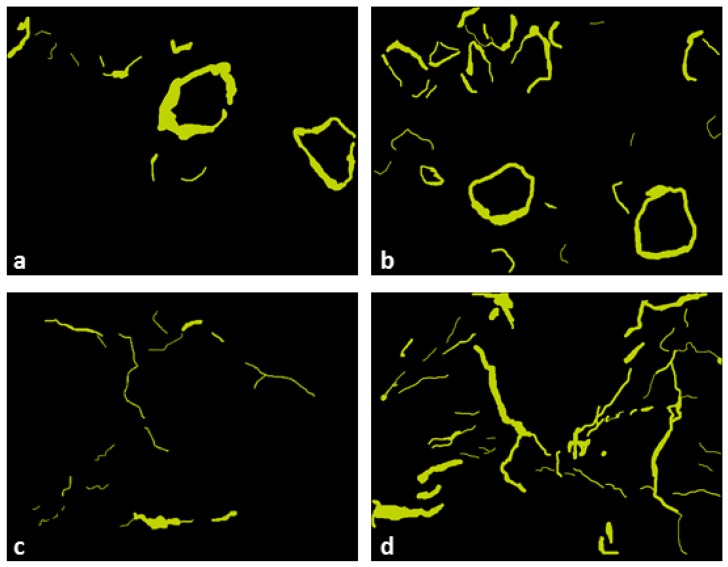
Crack porosity identification by the software Quantikov (**a**) crack porosity in clay with attapulgite fired at 800 °C, (**b**) crack porosity in clay with attapulgite fired at 1000 °C, (**c**) channels at clay with perlite fired at 800 °C and (**d**) channels at clay with perlite fired at 1000 °C.

In the samples with attapulgite, the formation of a crack porosity was observed, concentrating predominantly around the particles. Among the various quantified parameters, the main information refers to their corresponding area. In the sample fired at 800 °C, the area of the crack porosity represents 2.8% of the total, and at 1000 °C, 4.8%. The total area is also almost double (Table 1). These data prove the visual information, that the crack porosity increases greatly at 1000 °C (Figure 4b), when compared to the sample fired to 800 °C (Figure 4a). The width of this porosity is very variable, although it is visually estimated that there is an increase at 1000 °C, the appearance of new coronas around a larger number of narrower attapulgite particles masks this statistic.

The perlite was added to the base material in the proportion of 20% by weight. Measurements in samples fired at 800 °C and at 1000 °C represent 25% in area. Although the original perlite fragments are rounded, there is a large variation in the size of the particles whose diameters range from 262 to 3598 μm at 800 °C (Figure 3e) and 380 to 3293 μm at 1000 °C (Figure 3f). The degree of roundness of the preserved particles is 1, however, the average degree of rounding found in both samples is 0.77 (Table 1). These variations can be explained by the breaking of the particles during the transport and elaboration of the material. In the samples with perlite, the crack porosity with heating was also observed. It has an irregular shape, similar to micro channels, ranging from 18 to 225 μm in diameter at 800 °C, representing about 1.6% of the sample area. In the sample sintered at 1000 °C, the area of this porosity increases to 3.8% and the maximum width reaches 361 μm. The channels are more continuous, thicker and in greater proportion, as observed in the Figure 4c,d.

The grain size plays an important role in the onset of microcracking [34,35] which explains the microcracks generation in the ceramic with pore-forming agents since both atapulgite and perlite had much larger dimensions than the crystals or rock fragments found in the clay. The different shapes of crack porosity can be explained by the mismatch in anisotropic thermal expansion and elastic properties between randomly oriented grains during the cooling of polycristalline ceramics from the firing temperature generating microstresses along grain boundaries [33].

### 4.4. Morphology of Pores

**Figure 5:**
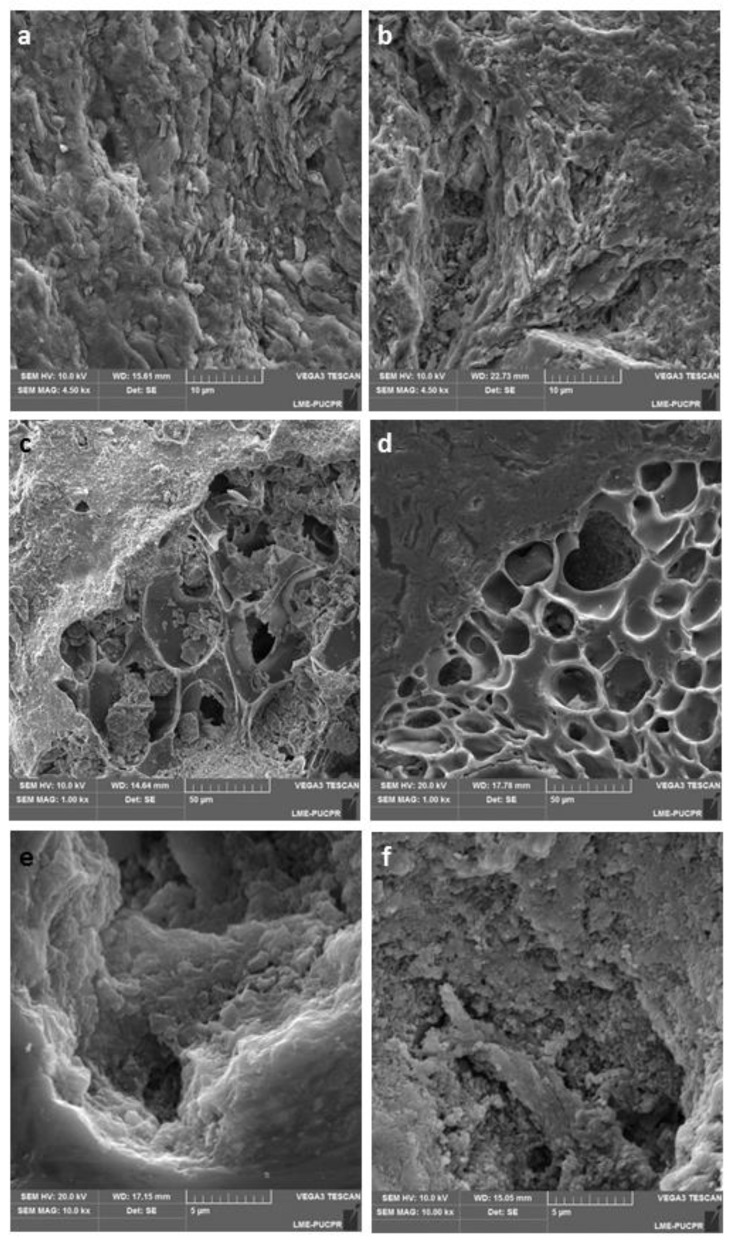
SEM images showing clay morphology at two different firing temperatures and 4500× magnification (**a**) 800 °C and (**b**) 1000 °C. Composition of perlite and clay at a magnification of 1000×: (**c**) 800 °C and (**d**) 1000 °C. Attapulgite with clay at a magnification of 10000×: (**e**) 800 °C and (**f**) 1000 °C.

The images obtained by electron microscopy show the shape and arrangement of the crystals, the granules and the primary porosity in the ceramic material (Figure 5). However, quantification of this porosity through image analysis proved to be much more difficult due to the random orientation of the crystals.

The morphological characteristics of the clayey base material show that the filossilicate leaves are still well defined in the sample fired to 800 °C (Figure 5a). Although the diffraction data showed that the kaolinite did not withstand the temperature rise and that only part of them were transformed to muscovite, the shape of the crystals was preserved. When compared to the images of the clayey material fired at 1000 °C (Figure 5b), there is a softening of the texture, the leaves of the phyllosilicates are less defined, they began to melt, indicating the decrease of the porosity with the increase of the temperature.

In the material with pore-forming agents, the matrix exhibits the same behavior of the clay base material with the heating, except for the generation of the crack porosity shown in Section 4.2. These absorbents have a much higher primary porosity than the clay base material, but with distinct characteristics. The perlite granules have a rounded, rough surface that is formed essentially of glassy material with bubbles, which often overlap. With the heating process, it was observed that the internal structure of the perlite granules in the sample at 800 °C (Figure 5c) are very similar to those of the original sample which is expected since perlite starts to melt above temperatures of 900 °C [19]. At 1000 °C (Figure 5d) the internal structure of the perlite is less homogeneous, the size of the bubbles decreases, the thickness of the edges of the bubbles is larger, part of the bubbles are filled with vitreous material and smooth portions are formed, evidencing the partial melting of the material.

The attapulgite granules are originally less rounded than the perlite ones, their surface is smoother with lamellar internal structure. The attapulgite granules in the sample sintered at 800 °C (Figure 5e) still preserve the lamellar texture, although the paligorskite is not stable anymore at this temperature. In the sample sintered at 1000 °C (Figure 5f), the internal texture of the attapulgite is predominantly fine granular, formed by short prisms smaller than 0.5 μm of length. This texture is consistent with the XRD data showing the predominance of quartz and microcline in attapulgite at the same temperature.

### 4.5. Pore Quantification

**Figure 6:**
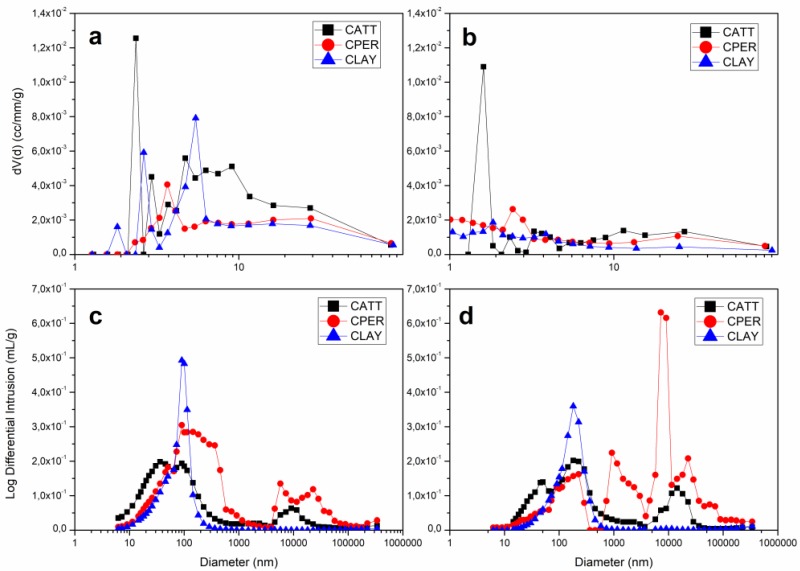
Pore size distribution from nitrogen adsorption test: (**a**) samples sintered at 800 °C and (**b**) samples sintered at 1000 °C and from mercury intrusion test: (**c**) samples sintered at 800 °C and (**d**) samples sintered at 1000 °C.

A comparison of pore size distribution (PSD) obtained from MIP and N${}_{2}$ adsorption experiments for samples sintered at 800 °C and 1000 °C shows a different trend when the pore-forming agents attapulgite and perlite are incorporated in clay and at two firing temperatures. The BET based technique was performed using nitrogen as the adsorbate at a bath temperature of 77.3 K. The relation between pore diameter and pore volume was determinated by the Barrett–Joyner–Halenda (BJH) method and illustrated in Figure 6a,b. It shows that, compared to other samples, the composition with attapulgite (CATT) has a microstructure with a greater number of small pores when it is sintered both at 800 °C and 1000 °C. However, for CLAY and CPER, increasing firing temperature decreases the number of small pores (Figure 6a,b). The surface area and the monolayer adsorption, originated from the adsorption isotherm, were calculated using the BET based method. Additionally, total pore volume and the mean mesopore diameter were estimated by the BJH analysis and all these data are summarized in Table 2. It can be seen that, clay surface area and total pore volume were increased when attapulgite and perlite were added to the base material for both firing temperature. Moreover, the average pore diameter, decreased for all samples when sintering temperature raises. Incorporating attapulgite and perlite to clay, diminishes the pore diameter value at 800 °C, but at 1000 °C, CPER exhibits a higher value of pore diameter than the one for clay. [36] found a positive correlation between pore diameter and pore volume but not with specific surface area for mesoporous silica suggesting that mesopore networks present different levels of corrugation. In this case, surface area and total pore volume are positively related counter to pore diameter. MIP results show that the clay base material has predominantly pores of 100 nm at 800 °C (Figure 6c). Raising firing temperature did not affect substantially clay pore size but diminished the number of pores of this diameter (Figure 6d). When perlite is added to clay and fired at 800 °C, the mixed material presents a microstructure with a bimodal pore distribution where diameters mostly at 10 nm and at 10 μm (Figure 6c). Figure 6d revels that increasing firing temperature, changes CPER pore distribution with diameters values situate mostly at 100 nm and in the interval from 1 μm to 100 μm. CATT has a bimodal pore distribution of mainly 100 nm and 10 μm at 800 °C and still has a bimodal distribution at 1000 °C, with an increment of the number of these pore groups.

The smaller pores measured by nitrogen adsorption (BET) were not observed by optical microscopy and, although some of them have been observed by electron microscopy, it was not possible to quantify them with the use of this technique. On the other hand, the mercury porosimetry data can be correlated to the microstructures studied. Samples show distinctly two groups of porosity: pores in the order of 10${}^{2}$ nm and pores of the order of 10${}^{4}$ nm. The pores of the first group can be correlated to porosity found in the three samples (clayey base material, clay with perlite and clay with attapulgite). The second group refers to the crack porosity, formed during heating process for the samples with the pore-forming agents, as observed in the optical microscopy images.

At 800 °C, in the sample with attapulgite addition, the first group of pores has a bimodal distribution, where the pores with smaller diameter are related to attapulgite granules and the pores with larger diameter refer to the clayey material of the matrix and are coincident with the peak of the clayey base material. In the sample with addition of perlite, the opposite occurs because the perlite porosity is much larger, as shown in Figure 6c,d.

In samples sintered at 1000 °C, there is a reduction of original porosity and the increasing of the crack porosity both with perlite and attapulgite addition to clay. Although the crack porosity area of the samples containing attapulgite, observed in the images and measured by Quantikov, is greater that the one found for the samples with perlite, the Hg porosimetry method shows that the amount of measured pores is higher in the sample with perlite than for the one with attapulgite. This can be explained by the shape of these crack porosities: the attapulgite corona-shaped pores are isolated, whilst the crack porosity of the sample with perlite is interconnected, facilitating the percolation of fluids.

## 5. Conclusions

This paper presented a detailed microstructural characterization of a clay material, a clay with addition of attapulgite and a clay with addition of perlite. Results were obtained for all samples at the firing temperatures of 800 °C and 1000 °C.

The following conclusions can be extracted from this study:

(a) The observation of the textures of the clay base material by optical and electronic microscopy showed that there was no generation of crack porosity with the firing temperature. The addition of attapulgite generated crack porosity in a shape of corona around the particles. Comparing materials sintered at 800 and at 1000 °C, there is an increase in the area and quantity of these coronas. The coronas shape, circular but isolated, will act as a closed porosity. In the sample with perlite addition, a crack porosity was generated in the form of channels, which increased in size and width with the increase of firing temperature, forming a network with high connectivity.

(b) The mineralogical study through XRD showed that there was variation of phases with the firing of the material. Attapulgite granules, initially formed by paligorskite, lizardite, muscovite and quartz are partially amorphized and the remaining minerals re-equilibrate. Paligorskita and lizardite disappear. At 800 °C there are still muscovite peaks, quartz and enstatite. At 1000 °C, the quartz and enstatite peaks predominate. Paligorskite is a mineral with high natural porosity and sorption capacity, hence, its disappearance or rebalancing for other mineral phases leads to a decrease in the primary porosity of the material. The amorphization observed in the diffraction occurs due the partial melting of the material, as observed in optical and electronic microscopy. Perlite granules showed evidence of partial melting and decreased internal porosity when sintered at 1000 °C.

(c) The generation of the crack porosity can be compared with the mercury porosimetry data. The increased interconnectivity of crack porosity in the samples with perlite at 1000 °C may explain the increase in pore size showed by the porosimetry test. In the sample with attapulgite, although the increase in crack porosity at 1000 °C is clearly observed in the pore group of greater size, the measured porosity is lower compared to one obtained by the addition of perlite at the same temperature. This behavior is due to the fact that this porosity remains isolated.

## Figures and Tables

**Table 1:** Quantification of the CATT, CPER and CLAY microstructural parameters.

Parameter		CATT 800 °C		CATT 1000 °C		CPER 800 °C	CPER 1000 °C	CLAY 800 °C	CLAY 1000 °C
		Grain	Corona	Grain	Corona	Grain	Grain	Grain	Grain
Area (μm${}^{2}$)	Total	2.32 × 10${}^{7}$	3.86 × 10${}^{6}$	2.66 × 10${}^{7}$	6.53 × 10${}^{6}$	1.09 × 10${}^{8}$	9.75 × 10${}^{7}$		
	Mean	2.90 × 10${}^{5}$	32,434	2.06 × 10${}^{5}$	27,681	8.11 × 10${}^{5}$	9.37 × 10${}^{5}$		
	Sdv	1.85 × 10${}^{5}$	51,439	1.76 × 10${}^{5}$	38,973	1.24 × 10${}^{6}$	5.89 × 10${}^{6}$		
	Max	8.06 × 10${}^{5}$	3.49 × 10${}^{5}$	9.53 × 10${}^{5}$	2.39 × 10${}^{5}$	9.71 × 10${}^{6}$	2.68 × 10${}^{6}$		
	Min	49,384	1133.6	20,476	318.8	51,581	94,340		
Diameter (μm)	Mean	591.16	170.7	484.01	163.88	886.17	1058.2	32.475	38.052
	Sdv	195.87	119.2	203.15	100.48	546.85	362.77	24.754	30.891
	Max	1036.5	681.86	1127.3	565.12	3598	1892.4	255	426.57
	Min	256.6	30.88	165.23	20.62	262.25	354.66	9.6624	9.6624
Roundness	Mean	0.74	0.43	0.74	0.39	0.77	0.77	0.69	0.71
	Sdv	0.087	0.15	0.083	0.1	0.084	0.071	0.096	0.094
	Max	1	0.78	1	0.84	1	1	1	1
	Min	0.52	0.14	0.52	0.15	0.57	0.58	0.44	0.44
Number of objects		80	119	129	236	137	104	856	1063
Objects/μm${}^{2}$		5.23 × 10${}^{-7}$	8.75 × 10${}^{-7}$	8.43 × 10${}^{-7}$	1.74 × 10${}^{-6}$	3.17 × 10${}^{-7}$	2.35 × 10${}^{-7}$	2.55 × 10${}^{-5}$	3.16 × 10${}^{-5}$
Fraction (%)		15.19	2.84	17.4	4.8	25.77	22.05		

**Table 2:** $N_{2}$ adsorption measurements for CATT, CPER and CLAY samples at different firing temperatures.

Sample	Specific Surface Area (m${}^{2}$/g)	Total Pore Volume (cm${}^{3}$/g)	Pore Diameter (nm)
CLAY 800 °C	18.3	0.12	5.46
CLAY 1000 °C	7.02	0.05	1.84
CATT 800 °C	29.53	0.16	2.36
CATT 1000 °C	11.94	0.09	1.61
CPER 800 °C	19.1	0.13	3.66
CPER 1000 °C	9.24	0.08	2.42
